# Common Combinations of Pregestational Diagnosis and Pregnancy Complications

**DOI:** 10.7759/cureus.19239

**Published:** 2021-11-03

**Authors:** Suzanne Cao, Fanglong Dong, C. Camille Okekpe, Inessa Dombrovsky, Guillermo J Valenzuela, Kristina Roloff

**Affiliations:** 1 Department of Women's Health, Arrowhead Regional Medical Center, Colton, USA; 2 Graduate College of Biomedical Sciences, Western University of Health Sciences, Pomona, USA

**Keywords:** adverse pregnancy outcomes, chronic disease, co-morbidity, multiple co-morbidities, pre-gestational diagnosis, pregnancy-related complications, obstetrical outcomes

## Abstract

Objective

Single pregestational diagnoses have been demonstrated to be associated with pregnancy-related complications. But, the effect of multiple diagnoses is understudied. The objective of this study is to determine the most common combinations of pregestational diagnoses and to determine if specific combinations increase the risk of pregnancy-related complications.

Study design

We performed a cross-sectional study of the 2016 Healthcare Cost and Utilization Project’s National Inpatient Sample (HCUP NIS) database. Inclusion criteria were: Diagnosis-related groups assumed to be associated with delivery, and three or fewer International Classification of Diseases, Tenth Revision (ICD-10), clinical modification codes with a prevalence greater than or equal to 0.5%, or clinically important risk factors in Bateman’s co-morbidity index. Chi-squared analysis of combinations of pregestational diagnoses was performed to assess the relative risk of pregnancy-related complications.

Results

The 2016 database included 255,233 delivered pregnancies. The most common combinations of pregestational diagnoses involved advanced maternal age, prior cesarean delivery, obesity, and tobacco use. Most combinations did not demonstrate an increased risk for complications greater than the risk with a single diagnosis. In those with statistically significant risk, all were 3-fold or less except we noted a 4.4-fold higher risk (95% CI: 3.16-6.15) of preeclampsia in obese patients of advanced maternal age compared to patients who were only of advanced maternal age.

Conclusion

Our results revealed that common combinations of pregestational diagnoses, in general, do not increase the risk for common pregnancy-related complications greater than the risk with a single diagnosis. This is reassuring, given that women entering pregnancy with multiple co-morbidities are becoming more common.

## Introduction

Intuitively, we hypothesized that pregnant women with multiple co-morbidities would experience more adverse pregnancy outcomes. Prior studies have demonstrated that isolated diagnoses are associated with pregnancy complications. For example, chronic hypertension is associated with an increased risk of preeclampsia, obesity with large for gestational age fetus, advanced maternal age with gestational diabetes, etc. [1-6]. But, the joint effect of multiple diagnoses is understudied.

The objective of this study was to determine the most common combinations of pregestational diagnoses and to determine if multiple diagnoses in common clusters increase the risk of pregnancy-related complications.

## Materials and methods

We performed a cross-sectional analysis of data from the Healthcare Cost and Utilization Project’s National Inpatient Sample (HCUP NIS) by the Agency for Healthcare Research and Quality (AHRQ) [7]. This administrative database is the largest hospital inpatient care database in the United States and includes all hospital discharges. The database includes a stratified sample of approximately 20% of all community hospitals and represents approximately 97% of the US population. We selected the 2016 database since this is the first full year of available data after the implementation of the International Classification of Diseases, Tenth Revision (ICD 10), Clinical Modification and the latest database available for research at the time this analysis was performed.

Inclusion criteria included women with: diagnosis-related groups (DRGs) assumed to be associated with delivery, and three or fewer ICD 10 codes. Women with ICD-10 codes that occurred within the database with less than 0.5% prevalence were excluded, under the assumption there would not be a meaningful contribution due to such low frequency. Because the process of selecting diagnosis codes by prevalence may have caused us to exclude some clinically important diagnoses, we referenced Bateman’s validated co-morbidity index, which is a list of maternal diagnoses associated with an increased risk of maternal morbidity and mortality [8]. Diagnoses included in this index with prevalence of less than 0.5% were added back into the analysis. Exclusion criteria were: DRGs assumed not to be associated with delivery, diagnoses with low prevalence, and women with missing data or four or more ICD-10 codes.

The following pregnancy-related DRGs are assumed by the authors to be associated with delivery: cesarean section with/without complication, vaginal delivery with/without complicating diagnoses, vaginal delivery with/without sterilization/dilation & curettage (D&C), postpartum and post-abortion diagnosis with/without procedure, and other antepartum diagnoses with/without medical complication. The following DRGs are assumed to not be associated with delivery: abortion with D&C, aspiration curettage or hysterotomy, ectopic pregnancy, threatened abortion, abortion without D&C, and false labor.

All ICD-10 codes with a prevalence greater than or equal to 0.5% within the database were analyzed and similar diagnoses were combined into clinically relevant categories. For example, type 1 and type 2 diabetes mellitus were grouped and categorized as diabetes mellitus. The ICD-10 codes were categorized as either a pregestational diagnosis or a pregnancy-related complication. Each diagnosis was reviewed by multiple authors, all maternal-fetal medicine physicians, and classified as either likely present prior to pregnancy (e.g., diabetes mellitus), or occurred during pregnancy (e.g., gestational diabetes mellitus). Table 1 lists the combined pregestational diagnoses and their associated ICD-10 codes and Table 2 lists the pregnancy-related complications. 

**Table 1:** Combined ICD-10 codes for pregestational diagnoses. ICD-10: International Classification of Diseases, Tenth Revision.

Pregestational diagnosis	ICD-10 code
Advanced maternal age (≥35 years old at the expected date of delivery)	O09.5x
Alcohol use*	F10.x
	O99.31x
	O35.4
	O35.4XXx
Asthma*	J44.x
	J45.x
Chronic kidney disease*	N02.x
	N03.x
	N04.x
	N05.x
	N18.x
	N25.x
	N26.9
	O26.83x
Congenital heart disease*	Q20.x
	Q21.x
	Q22.x
	Q23.x
	Q24.x
	Q25.x
	Q26.x
	O99.4x
Congestive heart failure*	I50.x
Cystic fibrosis*	E84.x
Diabetes mellitus	E08
	E10.x
	E11.x
	E13
	O24.0x
	O24.1x
	O24.3x
	O24.8x
	O24.9x
	R73.03
Fibroid uterus	D25
	O34.1x
HIV*	B20
	Z21
	O98.7x
Hypertension	I10
	I11.x
	I12.x
	I13.x
	I15.x
	O10.x
Ischemic heart disease*	I20.x
	I25.x
Mental health disorders (depression, bipolar, anxiety, schizophrenia, psychosis, etc.)	F06.3x
	F20
	F21
	F22
	F23
	F25
	F28
	F29
	F31.x
	F32.x
	F33.x
	F40.x
	F43.2x
	O99.3
	O99.34
	R45.85
Obesity	E66.x
	O99.21x
	Z68.3x
	Z68.4x
Prior cesarean delivery	O34.21
Pulmonary hypertension*	I27.x
Sickle cell disease/Thalassemia	D56.x
	D57.x
Substance abuse	F11.x
	F12.x
	F13.x
	F14.x
	F15.x
	F16.x
	F18.x
	F19.x
	O99.32x
	O35.5
Systemic lupus erythematosus*	M32.x
Tobacco use	F17.2x
	O99.33x
	Z87.7891
Valvular heart disease*	I05.x
	I06.x
	I07.x
	I08.x
	I09.x
	I34.x
	I35.x
	I36.x
	I37.x
	I38
	I39
*Prevalence less than 0.5% in NIS database, but included in Bateman’s co-morbidity index	

**Table 2:** Combined ICD-10 codes for pregnancy-related complications. ICD-10: International Classification of Diseases, Tenth Revision; HELLP: Hemolysis, Elevated Liver enzymes and Low Platelets; NIS: National Inpatient Sample.

Pregnancy-related complications	ICD 10 code
Chorioamnionitis	O41.12x
Gestational diabetes	R73.09
	R73.9
	O24.4x
	O99.81x
Hemorrhage (antepartum/intrapartum/postpartum)	D62
	O46.x
	O67.x
	O72.x
Infection (toxoplasmosis, parvovirus, cytomegalovirus, herpes simplex, rubella, viral, and parasitic)	B00
	B06
	B25
	B27.1
	B34.3
	B58
	O24.40
	O26.4x
	O35.3XXx
	O35.8
	O98.5x
	O98.6x
	O98.8x
	O98.9x
Intrauterine fetal demise	O36.4
	O36.4XXx
Intrauterine growth restriction	Z36.4
	O36.5x
Laceration (cervical, vaginal, perineal)	O70.x
	O71.3
	O71.4
Large for gestational age fetus	O36.6x
	P08.1
Malpresentation	O32.x
	O64.x
Meconium-stained amniotic fluid	O77.0
Non-reassuring fetal heart tones	O76
	O77.8
	O77.9
Oligohydramnios	O41.0x
Placental abruption	O45.x
Placental insufficiency	O36.51x
	O36.89x
Placenta previa*	O44.x
Polyhydramnios	O40.x
Preeclampsia (gestational, preeclampsia, eclampsia, HELLP syndrome)	O11.x
	O13.x
	O14.x
	O15.x
	O16.x
Preterm delivery	P07.3x
	O60.1x
Preterm premature rupture of membranes	O42.x
*Prevalence less than 0.5% in NIS database, but included in Bateman’s co-morbidity index	

Anemia was initially classified as a pregestational diagnosis. However, during analysis, we realized that the same ICD codes were being utilized in those patients with a postpartum hemorrhage. Therefore, we decided to exclude anemia as a pregestational diagnosis, as we could not confidentially isolate those patients who had anemia prior to pregnancy. To ensure that the database was a good representation of the population, we compared the prevalence of common pregnancy-related complications and single pregestational diagnosis to reference obstetric textbooks to ensure that they were similar (data not shown) [9,10]. 

Chi-squared analysis was performed to assess the relative risk of pregnancy-related complications for combinations of pregestational diagnoses compared to a single diagnosis. Statistical analysis was performed using SAS software, Version 9.4 for Windows (Cary, North Carolina, USA). A two-sided p-value <0.05 was considered to be statistically significant. Since this database is a limited data set with de-identified data, this study was exempt from Institutional Review [11]. 

## Results

The 2016 HCUP NIS database included 7,135,090 total patient discharges, of which 6,879,857 (96.42%) patients were excluded, mostly because they were not pregnant. Among those excluded, only 999 (0.01%) were excluded due to having four or more diagnoses. The final analysis consisted of 255,233 (3.58%) patients, who were assumed to be admitted for delivery with zero to three ICD-10 codes from our list (Tables 1 and 2). 

Most common combinations of pregestational diagnoses

We were interested in determining the most common combination of diagnoses. Table 1 lists 21 pregestational diagnoses, which means there is a total of 210 possible two diagnoses combinations and 1,330 possible three diagnoses combinations. Table 3 lists the most common combinations of two or three pregestational diagnoses. 

**Table 3:** Most common combinations of two or three pregestational diagnoses. Combinations with a prevalence of 0.1% or greater are shown. Total No. = 255,233.

	No. (%)
Two combination pregestational diagnoses	
Advanced maternal age and prior cesarean delivery	4,779 (1.87)
Obesity and prior cesarean delivery	1,920 (0.75)
Prior cesarean delivery and tobacco use	1,751 (0.67)
Obesity and tobacco use	723 (0.28)
Advanced maternal age and tobacco use	675 (0.26)
Advanced maternal age and obesity	659 (0.26)
Substance abuse and tobacco use	556 (0.22)
Hypertension and prior cesarean delivery	410 (0.16)
Advanced maternal age and hypertension	383 (0.15)
Hypertension and obesity	333 (0.13)
Advanced maternal age and fibroid uterus	257 (0.10)
Three combination pregestational diagnoses	
Advanced maternal age and obesity and prior cesarean delivery	327 (0.13)
Advanced maternal age and prior cesarean delivery and tobacco use	249 (0.10)

Of the 797,697 patients with a DRG assumed to be associated with delivery, 251,082 (31.55%) patients had one pregestational diagnosis, 114,411 (14.37%) had two pregestational diagnoses, and 42,604 (5.35%) had three pregestational diagnoses. The most common combinations of diagnoses included: advanced maternal age and prior cesarean delivery (4,779/255,233; 1.87%), obesity and prior cesarean delivery (1,920/255,233; 0.75%), and tobacco use and prior cesarean delivery (1,751/255,233; 0.67%). The results suggest that the most common combination of pregestational diagnoses involved advanced maternal age, prior cesarean delivery, obesity, and tobacco use, with the top six combinations involving some combination of these.

Relative risk of pregnancy-related complications for common combinations of pregestational diagnoses

In Table 4, we present results from a select combination of pregestational diagnoses.

**Table 4:** Relative risk of pregnancy-related complications in women with prevalent and clinically important combinations of pregestational diagnoses compared to those with either diagnosis alone.

	Gestational diabetes	Hemorrhage	Intrauterine growth restriction	Laceration	Large for gestational age fetus	Malpresentation	Meconium-stained amniotic fluid	No adverse outcome	Non-reassuring fetal heart tones	Pregnancy-induced hypertension
Advanced maternal age and hypertension No. = 383										
Advanced maternal age/Hypertension vs. Advanced maternal age No. = 14,459										
Relative risk	2.71	1.92	3.36	0.70	0.26	1.12	0.78	1.29	1.04	1.41
95% CI	1.85-3.95	0.61-6.09	1.47-7.66	0.60-0.82	0.04-1.86	0.68-1.84	0.29-2.07	1.09-1.53	0.63-1.72	0.70-2.82
Advanced maternal age/Hypertension vs Hypertension No. = 1,722										
Relative risk	2.79	1.77	3.14	0.93	0.47	1.77	1.57	0.84	1.09	0.62
95% CI	1.75-4.44	0.47-6.62	1.13-8.76	0.78-1.10	0.06-3.67	0.99-3.15	0.51-4.84	0.70-1.01	0.63-1.88	0.30-1.28
Advanced maternal age and obesity No. = 659										
Advanced maternal age/Obesity vs. advanced maternal age No. = 14,459										
Relative risk	2.12	0.87	0	0.72	2.66	0.96	1.05	1.12	0.80	4.41
95% CI	1.48-3.03	0.21-3.54	0	0.64-0.82	1.58-4.47	0.61-1.50	0.52-2.21	0.95-1.30	0.50-1.29	3.16-6.15
Advanced maternal age/Obesity vs. obesity No. = 5,818										
Relative risk	2.03	0.68	0	0.96	1.31	1.05	0.65	0.95	0.61	1.43
95% CI	1.39-2.97	0.16-2.85	0	0.84-1.10	0.77-2.23	0.66-1.67	0.32-1.32	0.81-1.11	0.38-0.99	1.03-1.99
Hypertension and obesity No. = 333										
Hypertension/Obesity vs hypertension No. = 1,722										
Relative risk	1.84	2.88	1.93	0.85	2.89	1.01	1.44	0.86	2.13	0.85
95% CI	1.02-3.30	0.88-9.51	0.53-7.05	0.70-1.03	1.00-8.37	0.46-2.23	0.41-5.07	0.70-1.04	1.37-3.33	0.43-1.69
Hypertension/Obesity vs. obesity No. = 5,818										
Relative risk	1.71	2.46	2.15	0.85	0.79	0.70	0.44	1.12	1.57	0.63
95% CI	1.00-2.91	0.87-6.95	0.65-7.09	0.70-1.03	0.33-1.92	0.33-1.47	0.14-1.38	0.92-1.35	1.05-2.32	0.33-1.21
Substance abuse and tobacco use No. = 556										
Tobacco use/Substance abuse vs. tobacco use No. = 8,789										
Relative risk	0.42	1.19	2.77	0.75	0.46	0.96	1.55	1.17	1.24	0.44
95% CI	0.13-1.30	0.43-3.26	1.80-4.26	0.65-0.87	0.06-3.36	0.55-1.67	0.95-2.53	1.05-1.30	0.87-1.76	0.18-1.06
Substance abuse/Tobacco use vs. substance abuse No. = 731										
Relative risk	0.78	1.30	2.00	0.82	0.43	0.81	1.58	1.17	0.88	0.38
95% CI	0.19-3.25	0.33-5.18	1.06-3.78	0.69-0.98	0.05-4.16	0.41-1.59	0.79-3.17	1.01-1.36	0.57-1.32	0.14-1.03

The authors reviewed the prevalence of a combination of pregestational diagnoses and determined clinically relevant ones for further analyses. Combinations reviewed included advanced maternal age, hypertension, obesity, prior cesarean delivery, tobacco use, and substance abuse. Although we were interested in looking at the effect of diabetes mellitus in combination with other diagnoses, the majority of diabetic patients (84%) were excluded from our analysis based on our established exclusion criteria (four or more ICD-10 codes or the presence of a low prevalence code). The number of patients with the diagnosis of diabetes mellitus in isolation was 0.1% (262/255,233). Due to a large number of possible combinations, the number of patients with diabetes mellitus in specific combinations (three or fewer ICD-10 codes) was even smaller. Due to this, comparative analysis of diabetes mellitus in combination with other pregestational diagnoses to a single diagnosis was not possible. In addition, the relative risk for only the most common pregnancy-related complications is presented here. Additional data is available at http://www.obgyndo.com/relative-risk-multiple-diagnoses.

In general, common combinations did not demonstrate an increased risk for complications compared to patients with a single diagnosis. An example is the addition of chronic hypertension when another pregestational diagnosis was present had no increased risk for preeclampsia compared to those with a single diagnosis. In combinations with statistically significant increased risk, all were three-fold or less except we noted a 4.4-fold higher risk (95% CI: 3.16-6.15) of preeclampsia in obese women of advanced maternal age compared to patients who were only of advanced maternal age. The relative risk also differed depending on which single diagnosis the combination was being compared. For example, in obese women of advanced maternal age, the risk for a large for gestational age fetus was 2.7-fold higher (95% CI: 1.58-4.47) compared to advanced maternal age alone, but not increased (RR: 1.3, 95% CI: 0.77-2.23) compared to obese patients not of advanced maternal age.

## Discussion

Surprisingly, we did not find a clinically significant increase in common complications in pregnant women with multiple diagnoses compared to patients with a single diagnosis. Women with multiple diagnoses had a minimal (three-fold or less), or no increased risk of complications compared to those with a single diagnosis. These results go against our clinical bias that more pregestational diagnoses may dramatically increase the patient's risk for complications. These results are reassuring and indicate that having multiple diagnoses may not have a compound effect on complications.

Our results also suggest that the most common combinations of pregestational diagnoses include modifiable or preventable conditions such as prior cesarean delivery, obesity, and tobacco use. This is important as nearly half of all pregnancies in the United States are unplanned [12,13]. A pregestational evaluation in women of reproductive potential may optimize pre-existing diseases in hopes of mitigating any negative impact on pregnancy outcomes. Women entering pregnancy with multiple co-morbidities are becoming more common [14]. While our results are reassuring, additional research on this growing population is still needed. 

The interaction of multiple diagnoses is complex and dynamic, and our results show the risk of complications differed depending on which single diagnosis was being used for comparison. For example, in patients with both obesity and tobacco use, there was an increased risk for intrauterine growth restriction only when compared to obese patients and an increased risk for large for gestational age fetus only when compared to tobacco users. It is not possible to elucidate the temporal relationship of these diagnoses given a cross-sectional study design and further research is needed.

The exclusion of patients with low prevalence diagnoses to reduce noise and produce a clean dataset is an important strength of this study. This strategy allowed us to isolate the impact of pregestational diagnoses and assess any synergistic effect when combined with another diagnosis. When patients with multiple diagnoses are compared to patients with a single diagnosis, any change in relative risk of complications is likely due to the additional diagnoses. Another strength of this study is our pragmatic approach. Given a large number of possible combinations, we made every attempt to isolate and present prevalent and clinically relevant diagnoses for analysis to provide results with practical implications that may influence a provider’s clinical practice. Another strength is the study sample size from the largest administrative database in the United States, utilizing ICD-10 codes, which has markedly expanded diagnostic accuracy compared with prior ICD versions due to the greater number of codes and increased specificity.

However, the use of an administrative database and ICD-10 codes is also a limitation of this study. Coded data are prone to under ascertainment bias leading to under-estimation [15-17]. Coded data are also subjected to misclassification bias, evident in the presence of ICD-10 codes for gestational diabetes among patients who already have diabetes mellitus. An administrative database is a record of hospitalization, so our analysis is confined to outcomes associated with delivery. Outcomes that were treated or resolved will not be reflected. In addition, as 2016 was the first full year of ICD-10 implementation, there is likely a learning curve among providers and coders. As the database is limited to the United States, the results may not be generalizable internationally.

Our inclusion and exclusion criteria methodology is another limitation of this study. As the number of possible combinations increased exponentially with additional diagnoses, we excluded patients with four or more diagnosis codes to help make the analysis more manageable. Even with three pregestational diagnoses, the number of combinations is large. This unfortunately may have decreased the prevalence rates of some diagnoses, such as diabetic patients, making some meaningful comparisons not possible. Another limitation based on our methodology is how we combined similar ICD-10 codes into a single category and categorized the diagnoses based on their assumed onset, either prior to or during pregnancy. Due to this, we lose the ability to determine if there is any association between individual pregestational subgroups with pregnancy-related complications (e.g., mental health combined depression, anxiety, bipolar disorder, etc.) or if there is any association between outcomes (e.g., gestational diabetes and risk for large for gestational age fetus, etc.) [18,19].

## Conclusions

In this study, we report the most common combinations of pregestational diagnoses and suggest that the presence of common clusters of diagnoses does not increase the risk of pregnancy-related complications compared to patients with a single diagnosis. The most common combinations of pregestational diagnoses involved conditions that are preventable or modifiable and may be corrected through early detection and pre-conception counseling. This is reassuring, given that women entering pregnancy with multiple co-morbidities are becoming more common.
